# Comparison Between Cultivation and Sequencing Based Approaches for Microbiota Analysis in Swabs and Biopsies of Chronic Wounds

**DOI:** 10.3389/fmed.2021.607255

**Published:** 2021-06-04

**Authors:** Aleksander Mahnic, Vesna Breznik, Maja Bombek Ihan, Maja Rupnik

**Affiliations:** ^1^National Laboratory for Health, Environment, and Food, Department for Microbiological Research, Maribor, Slovenia; ^2^Department of Dermatology and Venereal Diseases, University Medical Centre Maribor, Maribor, Slovenia; ^3^National Laboratory for Health, Environment, and Food, Department for Medical Microbiology, Maribor, Slovenia; ^4^Department of Microbiology, Faculty of Medicine, University of Maribor, Maribor, Slovenia

**Keywords:** chronic wound, *Corynebacterium*, wound microbiology, 16S amplicon sequencing, cultivation, swab, biopsy

## Abstract

Chronic wounds are a prominent health concern affecting 0.2% of individuals in the Western population. Microbial colonization and the consequent infection contribute significantly to the healing process. We have compared two methods, cultivation and 16S amplicon sequencing (16S-AS), for the characterization of bacterial populations in both swabs and biopsy tissues obtained from 45 chronic wounds. Using cultivation approach, we detected a total of 39 bacterial species, on average 2.89 per sample (SD = 1.93), compared to 5.9 (SD = 7.1) operational taxonomic units per sample obtained with 16S-AS. The concordance in detected bacteria between swab and biopsy specimens obtained from the same CWs was greater when using cultivation (58.4%) as compared to 16S-AS (25%). In the entire group of 45 biopsy samples concordance in detected bacterial genera between 16S-AS and cultivation-based approach was 36.4% and in swab samples 28.7%. Sequencing proved advantageous in comparison to the cultivation mainly in case of highly diverse microbial communities, where we could additionally detect numerous obligate and facultative anaerobic bacteria from genera *Anaerococcus, Finegoldia, Porphyromonas, Morganella*, and *Providencia*. Comparing swabs and biopsy tissues we concluded, that neither sampling method shows significant advantage over the other regardless of the method used (16S-AS or cultivation). In this study, chronic wound microbiota could be distributed into three groups based on the bacterial community diversity. The chronic wound surface area was positively correlated with bacterial diversity in swab specimens but not in biopsy tissues. Larger chronic wound surface area was also associated with the presence of *Pseudomonas* in both biopsy and swab specimens. The presence of *Corynebacterium* species at the initial visit was the microbial marker most predictive of the unfavorable clinical outcome after one-year follow-up visit.

## Introduction

Chronic wounds (CWs) are commonly defined as wounds that fail to spontaneously heal in 6 weeks (1) and are commonly classified into three most prevalent etiological categories: (1) venous valve insufficiency and dependency, (2) lower extremity arterial disease, and (3) diabetes (2). They affect ~2.21 per 1,000 individuals in the Western population, significantly reducing life quality of the patients and representing a costly burden for the health system (3).

Wound healing is a complex process, affected by various systemic and local factors, among which microbial burden is one of the major culprits for non-healing CWs (4). Colonization with bacteria and fungi has been previously linked to different CW specific parameters, temporal dynamics and healing outcomes (5–9). The impaired CW healing is partially associated with biofilm formation, which provides resistance against host defenses and antimicrobial therapy (10–12). For the guidelines on the appropriate biofilm sampling, characterization, treatment and monitoring of treatment effectiveness we refer the reader to Hoiby et al. (13). The role of individual bacterial species in CW development and healing process, however, remains largely unclear and results in frequent overuse and ineffectiveness of antimicrobial agents in the treatment of CWs (14, 15).

The diagnosis of CW infection currently relies on a combination of clinical judgment and microbiological cultivation of one of the potential specimens: swabs, which are non-invasive and more frequently used or wound tissue, obtained by a more demanding and invasive biopsy or curettage (16). Recent guidelines for CW biofilms specify tissue biopsies as a gold standard for microbiological diagnostics due to the sampling of both surface and deeper tissue (4).

Molecular approaches enabling the exact and cost-effective characterization of mixed microbial populations are slowly being integrated into microbiological diagnostics in general (17) and have been considered a tool for rutine diagnostics of CWs (18). Characterization of the microbiota in CWs is crucial in order to improve our understanding of its impact on the healing process. It is intuitive to assume that the molecular characterization, especially high-throughput sequencing, will outperform cultivation-based methods, because of the limitations associated with culturing the slow-growing and anaerobic microorganisms. However, only few studies up to date systematically evaluated the differences between the two approaches (19–22). Previous studies specifically performed on CWs used either sequencing-based methods (23) or cultivation-based methods (16, 24, 25) to compare between the swab and biopsy specimens.

In this study, we used sequencing- and cultivation-based approaches to analyze paired swab and biopsy specimens in order to evaluate different methodologies for characterizing the complex bacterial populations in CWs. Additionally, extensive metadata were used to reveal wound-specific and clinical-outcome-associated correlations with bacterial community structure.

## Materials and Methods

### Specimen Collection

The study included 45 in- and outpatients with CWs, who were treated in the Department of dermatology and venereal diseases at University Medical Center Maribor (Slovenia) from February to June 2017. The inclusion criteria were age above 18 years and CWs with duration of more than 6 weeks. Only one CW per patient was sampled. The ethical approval was obtained by institutional ethical board committee (UKC-MB-KME-23-13/17).

After obtaining patients' written informed consents, clinical data relating to the characteristics of the patients [age, gender, body mass index (BMI), and ankle-brachial index] and of the CWs (duration, estimated surface area calculated as length by width of the wound, topical and systematic therapy in previous month, clinical assessment of biofilm presence and clinical outcome of the wound after 1 year), were obtained. Although no generally accepted clinical signs of CW biofilms exist (26), extensive fibrinous slough has been proposed as possible macroscopic clue of CW biofilm (27) and this was also adopted in this study.

All CWs were cleaned with potable warm water, soap (pH 5.5), gauze and a round curette. The swabs were obtained according to the Levine technique, which consists of rotating a swab over 1 cm^2^ area with sufficient pressure to express fluid from within the CW tissue (24, 28). The swabs were placed in a sterile container with liquid medium and were sent within 24 h for downstream analysis. Next, 3–4 mm punch biopsies were performed on the same CW area under local anesthesia and the tissue samples were bisected, with half of the sample immediately frozen and stored in liquid nitrogen at −197°C until NGS, and the other half placed in sterile container and sent within 24 h for bacterial cultivation.

### Cultivation-Based Analysis of Samples

Swabs were re-suspended in the physiological solution. Suspension was divided in two aliquots, one of them was used for DNA isolation and sequencing, the other was initially inoculated into thioglycolate enrichment for 24 h. This was subsequently cultured on the blood agar and selective media for Gram negative bacteria. Plates were incubated either at 5% CO_2_ or at aerobic atmosphere for 24 h.

Tissue biopsies were initially homogenized in physiological solution (Millimix 20, DOMEL). The homogenate (100 μL) was transferred into thioglycolate broth and into cooked meat broth. After 24 h, enrichment cultures were inoculated on the same media and under same conditions as described above for swabs. Additionally, anaerobic cultivation was performed on COH and Schaedler agar for 48 h. Colonies were isolated in pure culture and identified with Maldi Biotyper (Bruker Daltonik).

### 16S Metagenomic Sequencing

The tissue biopsies and swab sample residues (in physiological solution) were stored at −80°C until molecular diagnostics. Total DNA was extracted with QIAamp DNA Mini kit (QIAGEN) with a modified protocol. Pellets were re-suspended in 360 μL of ATL buffer and homogenized [SeptiFast tubes (Roche), MagnaLyser (Roche), 7,000 rpm, 70 s]. Afterwards, 40 μL of proteinase K was added and the suspension was incubated at 55°C for 1 h. Next, 200 μL of AL buffer was added, followed by an incubation at 70°C for 30 min. After the addition of 200 μL of 96–100% ethanol, we transferred the content into column tubes and the subsequent steps followed the protocol provided in QIAamp DNA Mini kit. Extracted DNA was stored at −80°C until further use.

We sequenced the V3V4 variable region of the 16S rRNA gene. Libraries were prepared according to the 16S Metagenomic Sequencing Library Preparation (Illumina) protocol using the primer pair Bakt_341F (5′-CCTACGGGNGGCWGCAG-3′) – Bakt_805R (5′-GACTACHVGGGTATCTAATCC-3′), covering ~460 bp fragment length (29). Library quality was checked with Bioanalyzer High Sensitivity DNA Assay. Sequencing was performed on the Illumina MiSeq platform (2 × 300 bp, 5% PhiX).

### Sequence Data Analysis and Statistics

Quality filtering was performed using mothur software (30) with parameters as recommended by Kozich et al. (31). Alignment was performed using Silva reference base (Release 123). Chimeras were identified using mothur implemented UCHIME algorithm. Taxonomy was inferred with the RDP training set (v.12) (0.80 bootstrap value). We obtained a total of 7,576,427 reads (min: 14,453, max: 81,825, average per sample: 39460.56).

A negative control (dH_2_O) was included in every DNA isolation batch (14 samples), totaling 5 negative controls. High abundance of contaminants is expected in samples with low bacterial burden such as CWs. To eliminate as many contaminants as possible from our dataset we implemented the following procedure: for each operational taxonomic unit (OTU) we selected the negative control with the highest number of reads (*N*_max_); altogether 5 negative controls were used. Respective OTU was then removed from the sample if the number of reads was <5 × *N*_max._

Statistical analysis was done in mothur (alpha and beta diversity, Bray-Curtis dissimilarity) and in R using packages “vegan” and “ggplot2.” Analysis of sequencing reads at species taxonomical level for *Corynebacterium* genus was performed with Oligotyping tool (32) and BLAST (https://blast.ncbi.nlm.nih.gov/Blast.cgi; accessed May 05, 2020).

## Results

### Characteristics of Patients and Chronic Wounds

Patient cohort in this study consisted of 31 females (68.9%) and 14 males (31.1%). Patients were on average 73.6 years old (SD = 11.8), had an average BMI 31.8 (SD = 8.1) and ankle-brachial index 0.98 (SD = 0.18). All patients were treated with different topical agents while 9 patients (20%) also received systemic antibiotic therapy.

An average duration from initial diagnosis of CW up until sampling for this study was 48.6 months (SD = 82.2). Measured CW surface area was on average 5494.0 mm^2^, biofilm was clinically estimated in 28 CWs (62.2%). The majority of CWs were located on the lower legs and were classified into four etiological categories: venous/dependency (*n* = 30), mixed arterial-venous (*n* = 6), diabetic wounds (*n* = 2) and wounds of other etiologies (*n* = 7).

Complete metadata along with OTU-based community structure is available in Supplementary Figures 1, 2 for swabs and biopsy samples, respectively. Contingency table with absolute sequencing read count can be found in Supplementary Table 1.

### Cultivation-Based Analysis of Swabs and Biopsy Specimens

**Figure 1:**
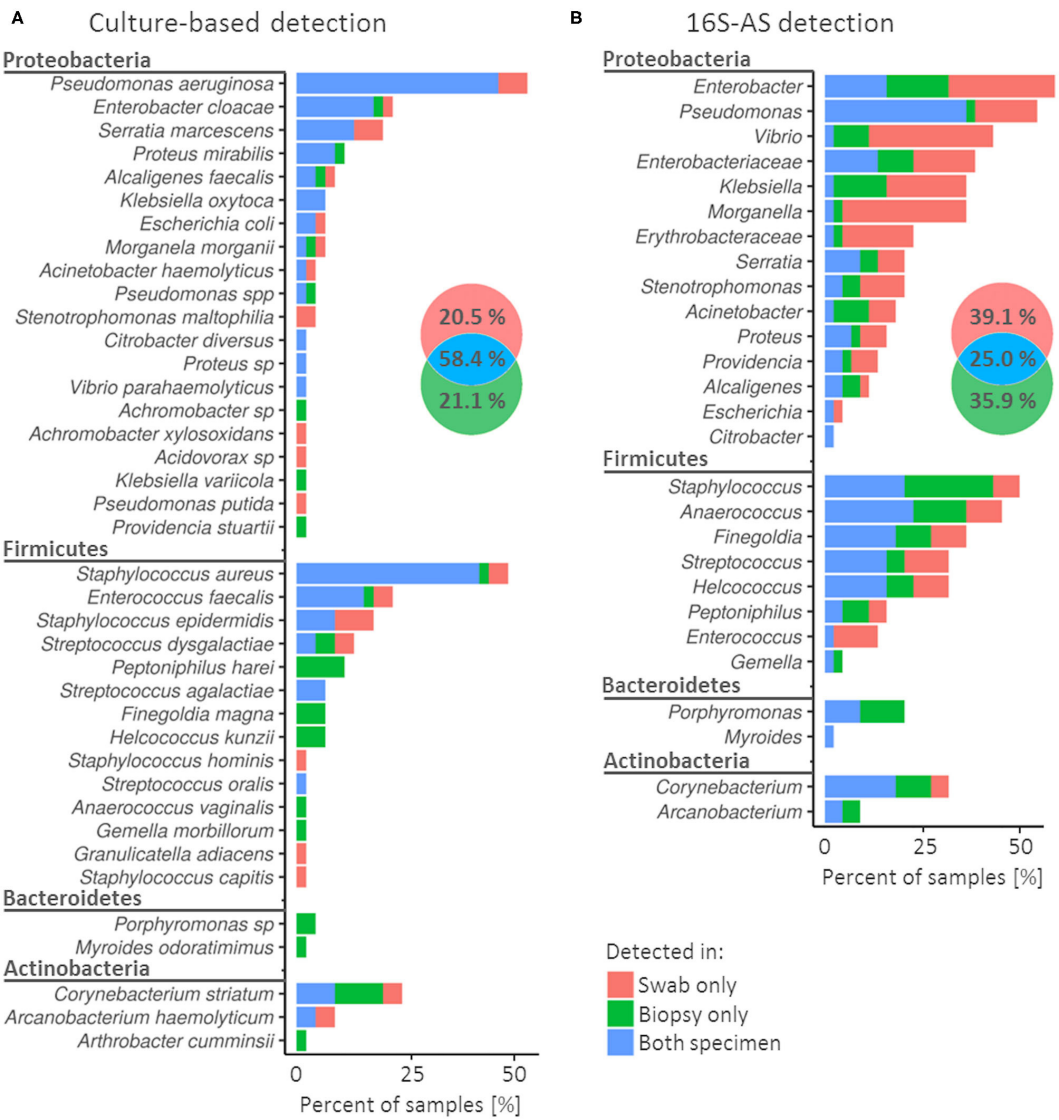
Concordance in the detection of bacterial species (cultivation) or genera (16S-AS) in swabs and biopsy specimens obtained from chronic wounds. Colors in the frequency histograms denote the percentages of samples according to the matching/non-matching detections between swab/biopsy sample pairs of the same CW. Results are presented for cultivation-based approach **(A)** and 16S-AS **(B)**, separately. Venn diagrams show cumulative percentages for all detected bacterial groups. Note that **(A)** (cultivation) represent diversity at the species taxonomic level while **(B)** (16S-AS) at the genus taxonomic level, therefore the direct comparison of detectable diversity between methods is not possible in this figure.

Swabs and biopsy specimens were collected from 45 CWs. Using cultivation approach, we detected a total of 39 different bacterial species, on average 2.89 species per sample (SD = 1.93). The average number of detected species per sample did not differ between biopsy and swab specimens (pairwise *t*-test, *p* = 0.93). The structure of bacterial population, however, showed only partial congruence between swab and tissue biopsy (Figure 1 and Supplementary Table 1). In 58.4% of cases, a respective bacterial species was detected in both specimens of the same CW, denoted hereinafter as a match. Highly prevalent bacterial species were more likely to match in swab/biopsy pairs of samples. For instance, when bacterial species was detected in at least five CW samples, we observed a 68% matching rate compared to the 37% matching rate in case of species that were detected in <5 CW specimens (Fisher exact-test, *p* = 0.027). In 41.6% of paired swab/biopsy samples we observed a miss-match, i.e., cases where a bacterial species was detected in only one of the paired swab/biopsy samples. Similar rates of miss-matches were observed for swabs (20.5%) and biopsy samples (21.1%) (Fisher exact-test, *p* = 1.000).

Cultivable bacterial community was represented by four bacterial phyla, of which *Proteobacteria* and *Firmicutes* showed the highest diversity and prevalence (Figure 1A). *Pseudomonas aeruginosa* and *Staphylococcus aureus* were the most prevalent species and were detected in 53% (24/45) and 49% (22/45) of CWs, respectively. In contrast, 43.6% (17/39) of detected species were each detected in only one CW sample (Supplementary Table 1).

When using cultivation approach, biased detection between specimens was most notable among the less prevalent bacterial species. For example, *Peptoniphilus harei* (*n* = 5)*, Finegoldia magna* (*n* = 3)*, Helcoccus kunzii* (*n* = 3) and *Porphyromononas* sp. (*n* = 2) were detected several times, but only in biopsy samples. On the other hand, *Stenotrophomonas maltophilia* (*n* = 2) was detected only in swabs (Figure 1A).

### Sequencing Based Analysis of Swabs and Biopsy Specimens

Amplicon sequencing of the V3V4 variable region of 16S rRNA gene (16S-AS) yielded 73 OTUs, on average 5.9 per sample (SD = 7.1). Similar to cultivation approach, the identified OTUs comprised four bacterial phyla, with *Proteobacteria* and *Firmicutes* dominating the complex communities (Figure 1B). Most prevalent were representatives from genera *Enterobacter, Pseudomonas* and *Staphylococcus*. 16S-AS analysis revealed culture-approach-associated under-representation of several taxa, mainly *Vibrio, Anaerococcus, Finegoldia* and *Enterobacteriaceae*.

The rate of miss-matches between swabs and biopsy samples was greater when using 16S-AS as compared to the cultivation-based approach (75 vs. 41.6%). This is likely a consequence of detecting bacteria, which are present in low bio-burden (Figure 1B). In both, culture- and 16S-AS-based approach, the detection of *Porphyromonas* was more consistent in biopsy specimens compared to swabs.

### Comparison Between 16S Amplicon Sequencing and Cultivation-Based Approach

**Figure 2:**
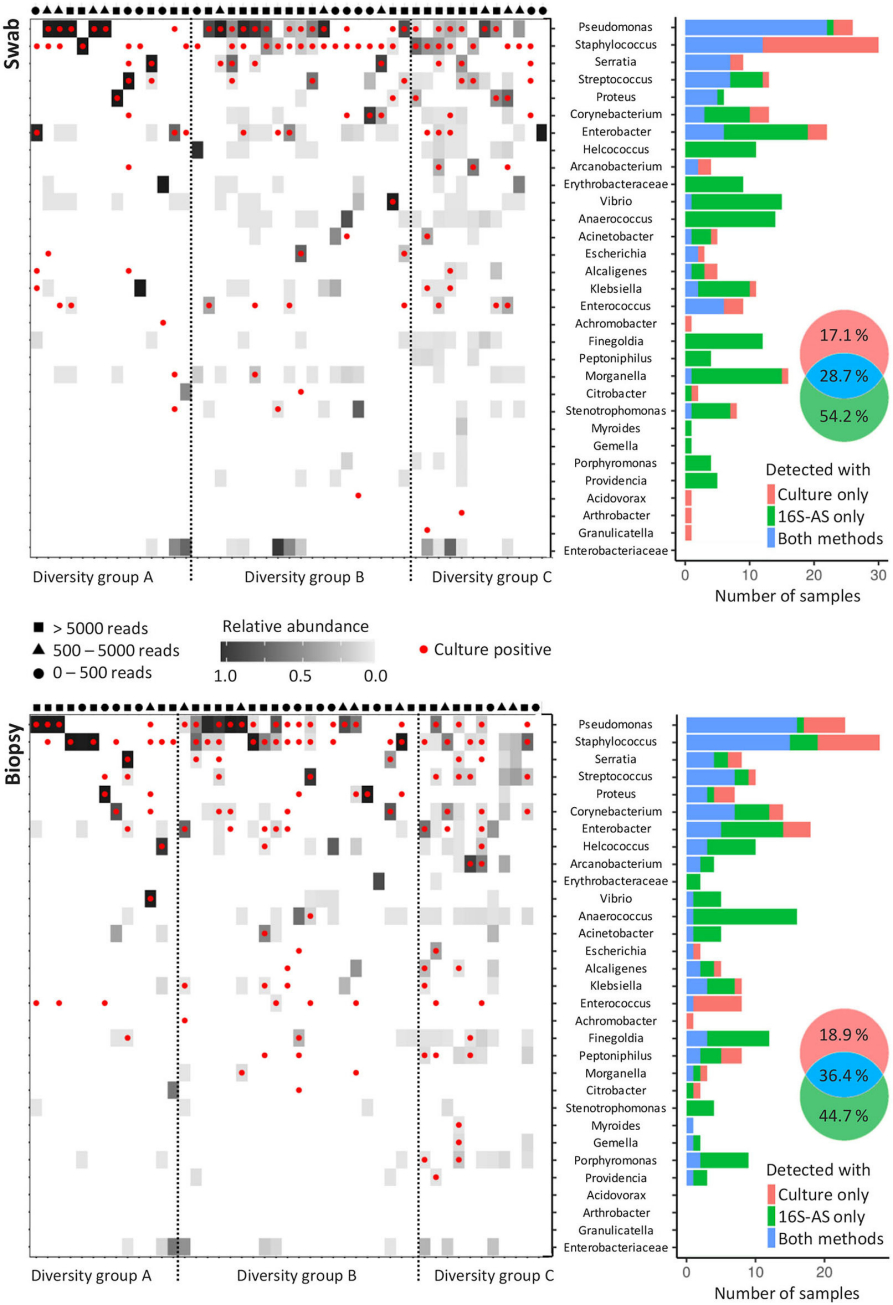
Concordance in the detection of bacterial genera with cultivation and 16S-AS in chronic wounds. Heat-plots show relative abundances of bacterial genera across all analyzed CW samples obtained from swabs (top) and biopsies (below). Red dots denote genera, which were also detected with cultivation-based approach. Samples were assigned to three diversity groups (A, B, and C), based on the number of operational taxonomic units (OTUs) that were required to cover 99% of total obtained sequencing reads. Diversity groups were not associated with the number of reads obtained per sample, indicated by the symbols shown above the heat-plots approximating the number of reads per sample into three categories. Histograms (right) show the concordance between cultivation-based detection and 16S-AS. Venn diagrams show cumulative percentages for all detected bacterial genera.

By using the sequencing data, CW samples were distributed into three groups based on the number of OTUs that were required to cover 99% of the obtained number of reads. Diversity group A included low diversity samples with a single OTU comprising 99% of all reads; diversity group B required 2 to 4 OTUs to cover 99% of obtained reads while the diversity group C required more than 4 OTUs (Figure 2 and Supplementary Figures 1, 2). Only 51.1% (23/45) swab/biopsy pairs were classified into the same diversity group.

The concordance between 16S-AS and cultivation-based detection of bacterial genera depended on the community diversity. Our results indicate that cultivation-based detection underestimates the bacterial richness in highly diverse bacterial communities (diversity group C) while no such bias was observed in case of low diversity communities (diversity group A) (Supplementary Figure 3). The concordance between 16S-AS and cultivation-based detection was higher in biopsy samples (36.4% matching rate) compared to the swab samples (28.7% matching rate) (Fisher exact-test, *p* = 0.002). Representatives from genera *Pseudomonas, Staphylococcus* and *Enterococcus* were most often detected with cultivation method while absent in 16S-AS data. Also, representatives from genera *Achromobacter, Acidovorax, Arthrobacter*, and *Granulicatella*, each detected once with cultivation, were not detected with 16S-AS. *Achromobacter* was the only one among these, which was removed from 16S-AS data due to the high number of reads found in the negative controls. On the other hand, several genera were often detected with 16S-AS, but not with cultivation. These included anaerobic representatives from *Anaerococcus, Finegoldia* and *Porphyromonas*; facultative anaerobes *Morganella, Vibrio* and *Providencia*, and aerobes from genera *Erythrobacteriaceae* and *Stenotrophomonas* (Figure 2).

### Microbiota Associations With Patient- and Wound-Specific Parameters and Clinical Outcome

In this study, underlying causes of the CWs included venous insufficiency/dependency, combination of peripheral arterial disease and venous insufficiency, diabetes and other causes (see Materials and methods). Due to the large disproportion in the number of samples of CWs from each etiological category, comparison between them was not possible.

Patient-specific factors (age, gender, and body mass index) were not significantly correlated with bacterial community structure neither in swab nor in biopsy specimens (Permutational multivariate analysis of variance (PERMANOVA) using Bray-Curtis distances, *p* ≥ 0.42; Supplementary Table 2). Bacterial community was not associated with antibiotic therapy (PERMANOVA, *p* > 0.78), while comparison between different topical treatments was not possible due to small number of cases in each subgroup.

**Figure 3:**
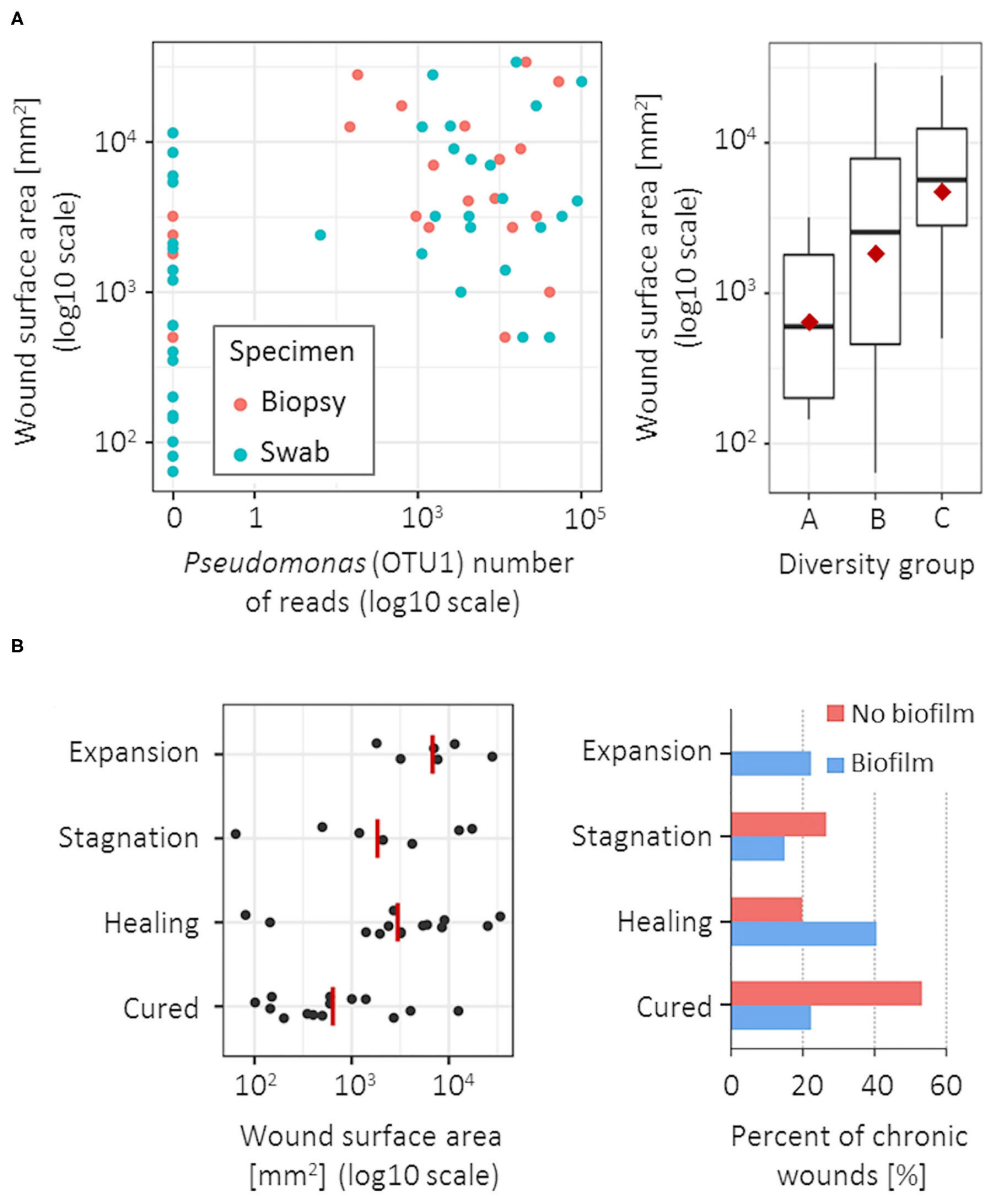
Microbiota association with wound surface area at the time of sampling and clinical outcome at 1-year follow-up. **(A)** CW surface area was positively correlated with the *Pseudomonas* (OTU1) abundance (left), significant for both biopsy (blue) and swab specimens (red); and with a larger diversity of the bacterial community, but only in swab specimens (right). **(B)** Unfavorable clinical outcomes were most significantly associated with a larger CW surface area (left) and the clinically estimated presence of biofilm (right). Red lines/points denote group means.

*Pseudomonas* (OTU1) was the only bacterial group significantly associated with a larger CW surface area, both in biopsy (Spearman's *r* = 0.55, *p* < 0.001) and swab specimens (Spearman's *r* = 0.48, *p* = 0.001; Figure 3A). The CW surface area was also positively correlated with the bacterial diversity in swab specimens (Spearman's *r* = 0.42, *p* = 0.008; Figure 3A), however no such correlation was observed in biopsy tissues. No correlation was found between bacterial community and CW duration or ankle-brachial index.

CWs were evaluated ~1 year after the sampling and were classified as cured, healing, stagnating or expanding. Higher abundance of *Corynebacterium* (OTU37 and OTU11) was associated with unfavorable clinical outcomes at the follow-ups, most commonly resulting in the stagnation of the healing process (Fisher exact-test, *p* = 0.092 and 0.072 for biopsy and swab specimen, respectively). Unfavorable clinical outcomes were also correlated with a larger CW surface area (Spearman's *r* = 0.44, *p* = 0.004) and the presence of biofilm at the time of CW sampling (Figure 3B). In the group of CWs with no clinical signs of biofilm, the recovery rate was 53.3% (8/15) compared to 22.2% (6/27) recovery rate of CWs with clinical signs of biofilm (Fisher exact-test, *p* = 0.085). Additionally, expansion of CW area on the follow-up was observed exclusively in cases with estimated biofilm at the first visit (6/45 CWs; 13.3%). In CWs with clinically estimated biofilm, microbiota showed slightly lower richness (3.2 OTUs per sample), compared to biofilm-free CWs (5.3 OTUs per sample) (*p* = 0.027); however, no specific bacterial groups could be correlated to the biofilm formation.

Additionally, we observed a strong co-occurrence between five OTUs comprising *Peptoniphilus* (OTU14 and OTU33), *Finegoldia* (OTU18) and *Anaerococcus* (OTU21 and OTU24) (mean Spearman's *r* = 0.59). At least two of these five OTUs co-occurred in 15/45 CWs (33.3%) and all five co-occurred in 3/45 samples (6.7%) (Supplementary Figure 4).

## Discussion

The aim of the present study was to compare different approaches for characterization of bacterial communities residing in CWs. We evaluated the differences between swabs vs. biopsy specimens and differences between performing 16S amplicon sequencing (16S-AS) vs. cultivation-based methods. Finally, we associated microbiota signatures with patient- and wound-specific parameters and clinical outcomes at the 1-year follow-up.

Comparison between swabs and tissue samples did not reveal significant advantage of one method of sampling over the other which is in concordance with previously reported studies (23, 25). However, we observed that the rate of concordance between cultivation and 16S-AS was higher when analyzing biopsy tissue (36.4%) compared to the swab samples (28.7%). Concordance between the swab/biopsy pairs of the same CW was 58.4 and 25.0% for cultivation-based and 16S-AS analysis, respectively. The relatively low matching rate was likely a consequence of the detection of bacteria present at low cell concentration and sampling bias due to the heterogeneity of bacterial communities along wound surface (33) and inside biofilms (34).

Previous studies already demonstrated the advantages of using sequencing-based methods over cultivation for the characterization of bacterial communities in CWs (19, 21, 22). In this study, the 16S-AS approach appeared advantageous over cultivation when characterizing communities with high bacterial diversity. In up to 60% of CWs with the highest bacterial diversity (more than 4 OTUs presented 99% of total obtained sequencing reads), the respective genera were detected solely with 16S-AS. On the other hand, in the low diversity samples dominated by a single bacterial group we did not observe any advantage of one method over the other. The majority of bacterial groups that we failed to detect with cultivation, included obligate and facultative anaerobic bacteria from genera *Anaerococcus, Finegoldia, Porphyromonas, Morganella* and *Providencia*, which is in concordance with previous publications (19, 21). Interestingly, the five OTUs corresponding to three Gram-positive anaerobic cocci (*Anaerococcus, Finegoldia*, and *Peptoniphilus*) frequently co-occurred in our study, forming a consortium which has already been reported (35), however its clinical relevance remains to be elucidated.

In our study, biofilm was clinically diagnosed in 62% of CWs, which is less frequent compared to the reported rates of at least 78% of CWs (36). Biofilms have prominent influence on development of CWs and hamper CW healing due to the increased resistance against the host defenses and antimicrobial therapy (10, 11). In this study, CWs with clinically assessed biofilm at the time of sampling, showed 31% lower recovery rate compared to those without estimated presence of biofilm. Additionally, all the CWs, which showed the surface area expansion at 1-year follow-up, had a diagnosed biofilm at the first visit. It has been reported that CWs with clinically assessed biofilms were associated with a larger surface area, which is an established predictor of impaired CW healing (37, 38). In this study, only 15% of CWs with a surface area larger than 1,000 mm^2^ healed in a period of 1 year. Microbiota association analysis revealed that larger CW surface area at the time of sampling positively correlated with an increased abundance of *P. aeruginosa* and higher bacterial diversity and, the latter being in disagreement with previous study by Loesche et al. (8), which reported improved healing of diabetic foot ulcers with higher bacterial diversity.

Increased abundance of *Corynebacterium* was the single most predictive bacterial marker associated with unfavorable clinical outcomes in our study. *Corynebacterium* species are generally perceived as commensal (39); and the abundance of *Corynebacterium* was shown to be inversely correlated with *S. aureus*, suggesting potential protective role (40). However, under the right circumstances, *Corynebacterium* species can be clinically relevant (41–43) and the targeted treatment against this bacterium has shown improvement in the CW healing process (44). In this study, only *Corynebacteirum striatum* was identified by cultivation, corresponding to the more prevalent OTU11 in the 16S-AS dataset. However, an additional less prevalent *Corynebacteirum* OTU37 was also associated with unfavorable clinical outcomes. Top BLAST hits suggested OTU 11 and OTU 37 to belong to either *C. pseudodiphtheriticum* or *C. propinquum*. Reports on the clinical relevance of either of these two *Corynebacterium* species in the context of CW infections are rare (45, 46).

In previous reports, *Staphylococcus* has been reported as the most prevalent genus present in the microbiota of CWs, besides *Pseudomonas* (9, 47), however no correlation between *Staphylococci* and CW-specific factors or clinical outcome were found in this study.

A limitation of this study is that after obtaining the wound samples, the patients were not followed up thoroughly in terms of recording topical and systemic antimicrobial treatment, compression treatment with its specification, mobility, chronic and acute co-morbidities, especially peripheral arterial disease, anemia and nutrition. For appropriate correlation analysis of wound outcome, all these factors should also be addressed. Another limitation of our study is the small number of samples. Community composition differed greatly among CWs therefore larger sample sizes will be required in the future to improve the significance of correlations with clinical data. Also, the most common sequencing methodology (16S-AS) has an inherent limitation, as it does not enable characterization of microbiota at the species level, which would again better elucidate the clinical relevance of the observed trends.

In conclusion, the comparative analysis of 45 CWs included in this study showed that swabs and biopsy tissues are comparably sufficient for the correct identification of the dominating bacterial colonizers in CWs. Sequencing based approach was more efficient at capturing the broad spectrum of bacteria in communities with high bacterial diversity, detecting multiple additional obligate and facultative anaerobic bacterial taxa. However, currently sequencing is not a time- and cost-effective method, ready to be introduced in the routine diagnostics. The main strength of the method is identification of potential bacterial markers for unfavorable clinical outcome, as shown in our study for Corynebacterium species.

## Data Availability Statement

The datasets presented in this study can be found in online repositories. The names of the repository/repositories and accession number(s) can be found below: NCBI BioProject, Accession No: PRJNA659504.

## Ethics Statement

The studies involving human participants were reviewed and approved by University Medical Center Maribor institutional ethical board committee. The patients/participants provided their written informed consent to participate in this study.

## Author Contributions

AM has performed sequencing, data analysis, and was major contributor in writing the manuscript. VB has provided samples, clinical data, contributed to project design and operation, data interpretation, and manuscript preparation. MB performed cultivation tests and contributed to project design, and operation. MR contributed to project design and operation, data interpretation, and was major contributor in writing the manuscript. All authors read and approved the final manuscript.

## Conflict of Interest

The authors declare that the research was conducted in the absence of any commercial or financial relationships that could be construed as a potential conflict of interest.
