# Supplementary material for: Comparison Between Cultivation and Sequencing Based Approaches for Microbiota Analysis in Swabs and Biopsies of Chronic Wounds
Source: Front Med (Lausanne). 2021 Jun 4;8:607255. doi: 10.3389/fmed.2021.607255 (PMC8211761; doi:10.3389/fmed.2021.607255)
Supplement: Supplementary file 2 [file Data_Sheet_1.PDF]

Supplementary Material

Supplementary Table S2: Correlation between host specific factors and the composition of bacterial microbiota on wounds. Permutational analysis of variance (PERMANOVA, 1000 permutations) results are shown for swab (above) and biopsy specimen (below) separately.

	**Swab**		**Biopsy**	
	R2	p Value	R2	p Value
**Age**	0.02033	0.518	0.02047	0.578
**Gender**	0.02279	0.420	0.00866	0.994
**BMI**	0.01772	0.646	0.01150	0.933
**Systemic antibiotic treatment**	0.01455	0.782	0.01345	0.823

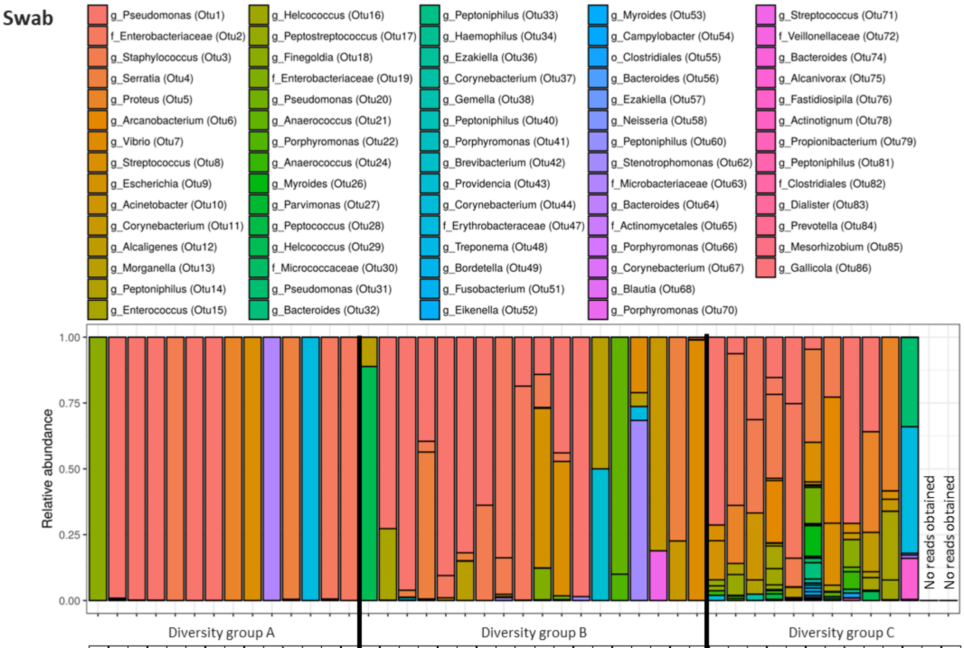

Diversity group	Clinical update after 1 year or less	Age [years]	Gender [M/F]	BMI	Topical therapy	Systemic antibiotic therapy	Duration [months]	Wound area [mm²]	Ankle index	Clinical estimation of bacterial burden	Clinical estimation of biofilm presence
A	Healing	70	M	42.6	Alginate with silver	None	12	144	NA	Colonized	Yes
A	Cured	84	F	31.2	Povidone iodine gauze	None	24	1000	0.9	Critically Colonized	Yes
A	Stagnation	77	F	34.9	Alginate with silver	None	14	NA	1.26	Colonized	No
A	Healing	51	F	59.5	Hydrofiber with silver	None	6	3200	0.8	Critically Colonized	Yes
A	Cured	66	M	35.2	Betamethasone/gentamycin	None	4	144	0.94	Infected	Yes
A	Expansion	79	F	27.2	Alginate with silver	None	24	1800	NA	Critically colonized	Yes
A	Expansion	31	M	40.8	Dry gauze	None	120	3200	1.24	Critically colonized	Yes
A	Cured	84	M	22.1	Betamethasone/gentamycin	None	3	NA	0.7	NA	NA
A	Cured	75	F	42.2	Hydrofiber with silver	None	120	400	1	Colonized	No
A	Stagnation	70	F	33.7	Dressing with silver	None	6	NA	1.18	Colonized	Yes
A	Cured	60	F	36.1	Fusidic acid cream	None	3	200	0.98	Colonized	No
A	Cured	68	F	30.1	Hydrofiber with silver	Moxifloxacin	5	600	1	Critically colonized	Yes
A	Cured	87	M	22.6	Petrolatum gauze	None	1	100	0.86	Colonized	No
A	NA	73	F	40.4	Alginate with silver	Ciprofloxacin	2	NA	0.9	Critically colonized	Yes
B	Cured	84	F	25.8	Octenidine dihydrochloride gel	Penicillin	3	150	0.81	Colonized	Yes
B	Healing	60	F	NA	Alginate with silver	None	13	34000	1	Critically colonized	Yes
B	Healing	65	M	33.2	Hydrofiber with silver	None	120	9000	1.04	Critically colonized	Yes
B	Stagnation	77	F	24	Alginate	None	12	500	0.6	Critically colonized	Yes
B	Healing	69	M	39.9	Povidone iodide gauze	None	2	25200	NA	Infected	Yes
B	Cured	68	F	34.1	Hydroactive dressing with Ringer	Amoxiclav	10	4050	1.04	Infected	Yes
B	Healing	82	F	25.1	Alginate	None	7	2700	0.77	Critically colonized	Yes
B	Stagnation	82	M	23.2	Zinc oxide bandage	None	120	1200	1.11	Critically colonized	Yes
B	Expansion	86	F	20.2	Povidone iodide gauze	None	120	7650	1.27	Colonized	Yes
B	Cured	80	F	32.2	Dry gauze	None	12	1400	1.02	Colonized	No
B	Expansion	99	F	26.4	Povidone iodide gauze	None	12	7000	1.1	Infected	Yes
B	Stagnation	87	M	23.5	Foam	None	36	4200	1.18	Critically colonized	No
B	Cured	64	F	43.3	Alginate	None	120	12600	0.75	Critically colonized	Yes
B	Healing	59	F	51.3	Fusidic acid cream	Penicillin	2	80	0.8	Colonized	No
B	Healing	76	F	30.1	Hydrofiber with silver	None	12	8500	1	Critically colonized	Yes
B	Cured	56	F	25.7	Betamethasone/gentamycin	Carbamazepine	1	350	NA	Colonized	No
B	Healing	78	F	30.5	Hydrofiber with silver	None	480	1950	0.89	Critically colonized	Yes
B	Stagnation	81	F	NA	Dressing with silver	None	13	63	1.13	Critically colonized	No
C	Healing	81	F	28.5	Silicone foam with silver	None	25	2400	1.09	Critically colonized	Yes
C	Cured	87	M	24.8	Alginate	None	9	2700	NA	Colonized	No
C	Healing	80	F	31.8	Alginate with silver	None	14	3200	1.15	Critically colonized	No
C	Expansion	87	F	24.5	Hydrofiber with silver	None	NA	11475	0.6	Critically colonized	Yes
C	Cured	64	F	33.8	Alginate with silver	None	120	500	1.11	Colonized	No
C	Stagnation	83	F	33.2	Dry gauze	None	120	12750	NA	Colonized	No
C	Cured	69	F	25.6	Hydrocolloid dressing	None	120	600	0.99	Critically colonized	No
C	Expansion	72	M	28.7	Octenidine dihydrochloride gel	None	24	28000	0.9	Infected	Yes
C	Stagnation	76	F	34.9	Foam	Amoxiclav	12	17400	NA	NA	NA
C	Healing	72	F	33	Silver sulphadiazine cream	NA	120	5950	1.23	Critically colonized	Yes
C	Healing	73	M	26.5	Neomycin ointment	Azithromycin	2	5400	NA	Critically colonized	No
C	Healing	65	M	NA	Hydrofiber with silver	None	14	1400	NA	Critically colonized	Yes
C	Stagnation	77	M	26.9	Silver sulphadiazine	Penicillin	120	2100	0.95	Critically colonized	Yes

Row groups in the figure: Patient metadata (Age, Gender, BMI); Patient treatment 1 month before CW sampling (Topical therapy, Systemic antibiotic therapy); Wound characteristics (Duration, Wound area, Ankle index); Clinical status (Clinical estimation of bacterial burden, Clinical estimation of biofilm presence).

Supplementary Figure S1: OTU-based community structure and metadata for swab samples. The stacked bar-plot presents the OTU-based community structure intendent primarily to show the differences between observed between the three diversity groups. For each sample the available host-specific factors, wound parameters and clinical information are listed. Diversity groups are based on sequencing data and the number of OTUs that represent 99 % of all sequence reads per sample (group A 1 OUT; Broup B 2-4 OTUs; group C more than 4 OTUs).

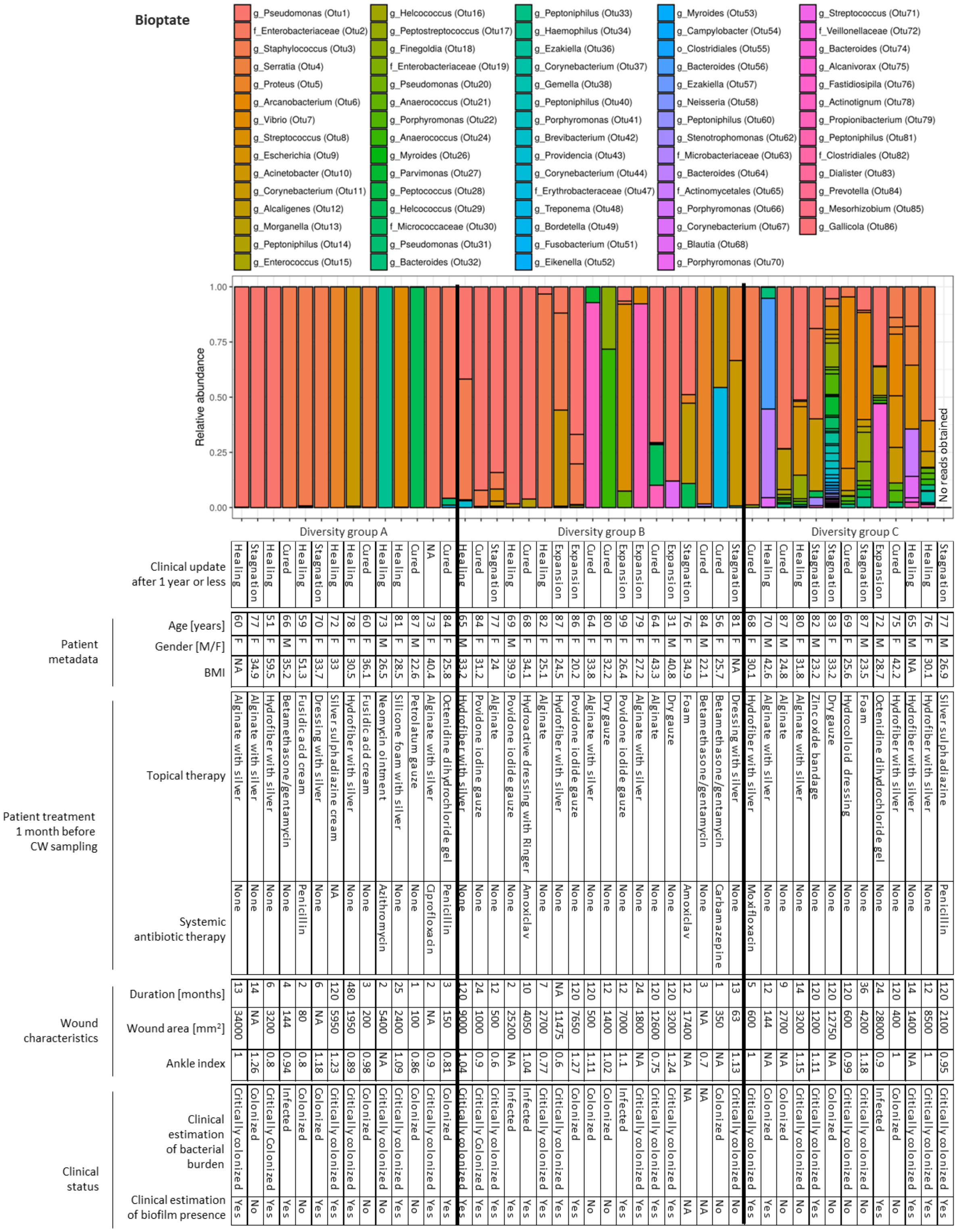

Supplementary Figure S2: OTU-based community structure and metadata for biopsy samples. The stacked bar-plot presents the OTU-based community structure intendent primarily to show the differences between observed between the three diversity groups. For each sample the available host-specific factors, wound parameters and clinical information listed. Diversity groups are based on sequencing data and the number of OTUs that represent 99 % of all sequence reads per sample (group A 1 OUT; Broup B 2-4 OTUs; group C more than 4 OTUs).

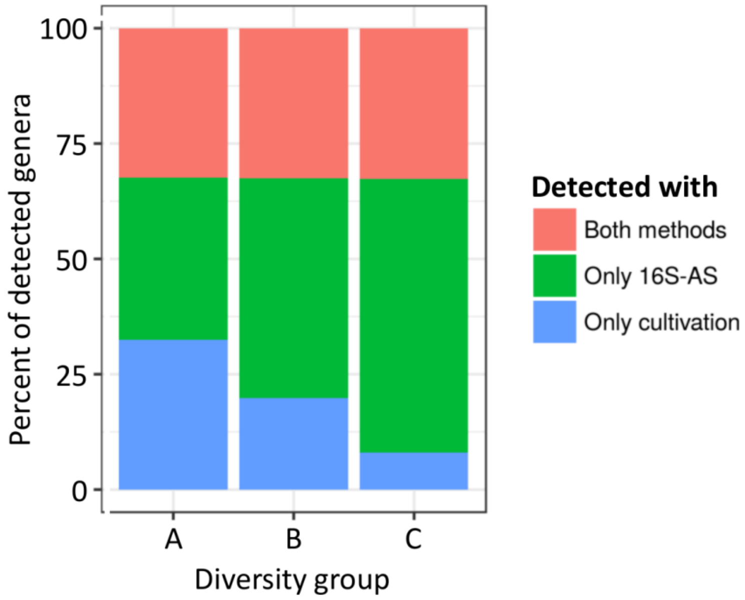

Supplementary Figure S3: Concordance in the detected bacterial genera between culture-based approach and 16S amplicon sequencing (16S-AS) according to the diversity group. Diversity groups were defined based on the number of OTUs that were required to cover 99 % of the obtained number reads. Diversity group A includes low diversity samples where a single OTU comprises 99 % of all reads. In diversity group B 2 - 4 OTUs cover 99 % of total reads. In diversity group C more than 4 OTUs cover 99% of total reads. Colours in the histogram denote the percent of detected genera (cumulative value for all samples) which matched between the cultivation-based approach and 16S-AS (red). The percent of miss-matches when the respective genus was detected only with culture is denoted in blue while the percent of miss-matches when the genus was detected only with 16S-AS is denoted in green. Most prominently, when bacterial community increased in diversity (from group A to C), increasingly more genera were detected only with 16S-AS method (green). Interestingly, percent of matches (red) was not affected by community diversity.

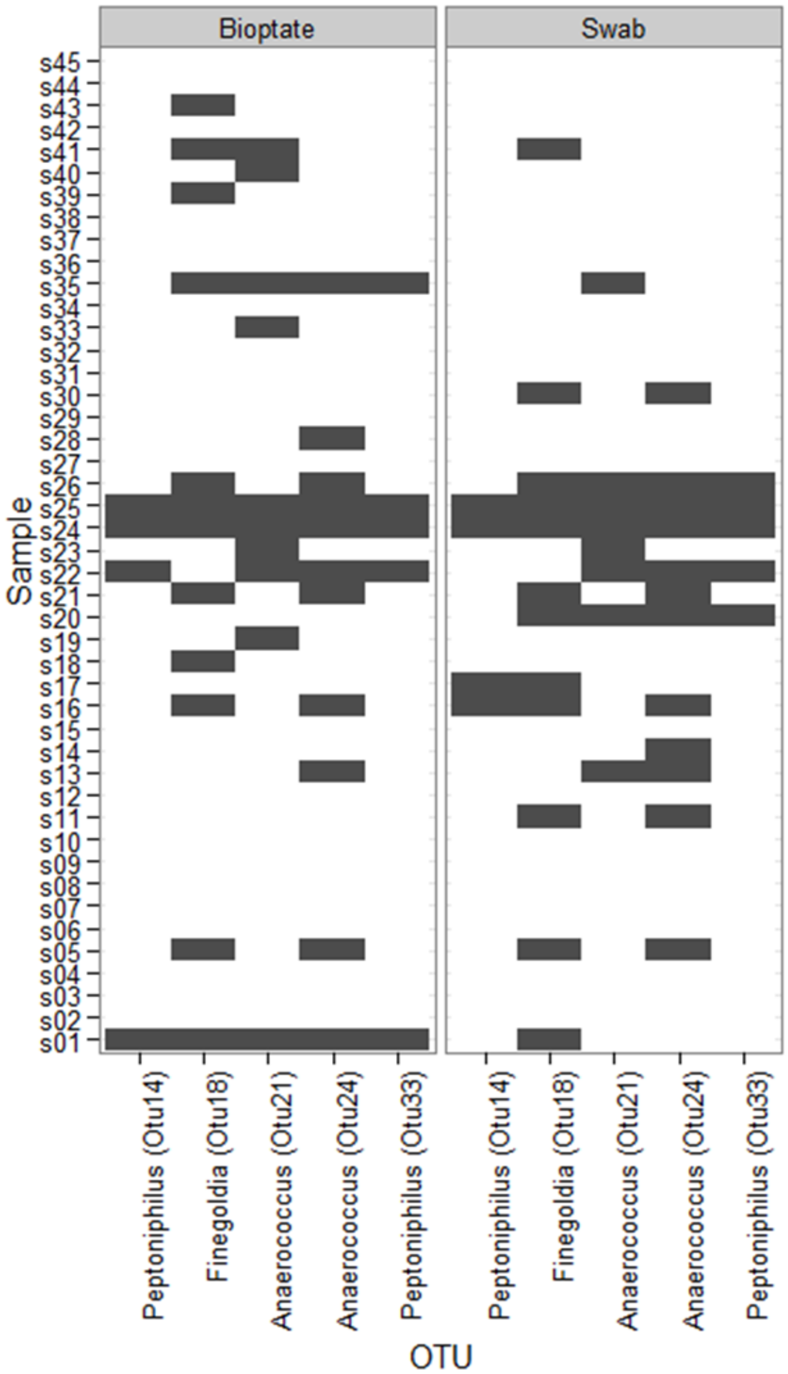

Supplementary Figure S4: Co-occurrence of five OTUs. Co-occurrence between OTUs was checked with Spearman's test. Darkened brackets denote the presence of five OTUs that frequently co-occurred in all 45 wounds for biopsy (left) and swab (right) separately. At least two of these five OTUs co-occurred in 15/45 wounds (33.3 %) and all five co-occurred in 3/45 samples (6.7 %).
